# 放疗时间选择对局限期小细胞肺癌预后影响的*meta*分析

**DOI:** 10.3779/j.issn.1009-3419.2010.09.10

**Published:** 2010-09-20

**Authors:** 辉 赵, 俊东 谷, 峰 滑, 洪瑞 许, 莉 李, 炳军 杨, 友奎 韩, 树忠 刘, 松超 洪

**Affiliations:** 1 300121 天津，天津市人民医院胸外科 Deapartment of Thoracic Surgery, Tianjin People's Hospital, Tianjin 300121, China; 2 300052 天津，天津医科大学总医院，天津市肺癌研究所，天津市肺癌转移与肿瘤微环境重点实验室 Tianjin Key Laboratory of Lung Cancer Metastasis and Tumor Microenvironment, Tianjin Lung Cancer Institute, Tianjin Medical University General Hospital, Tianjin 300052, China

**Keywords:** 肺肿瘤, 放疗, *meta*分析, Lung neoplasms, Radiotherapy, *meta*-analysis

## Abstract

**背景与目的:**

研究结果显示序贯化放疗是小细胞肺癌主要的治疗手段，它可以提高患者的生存率，但是何时开始实施放疗仍然存在争议。本研究旨在探讨放疗实施时间对局限期小细胞肺癌预后的影响。

**方法:**

通过计算机检索Medline、CENTRAL(the Cochrane central register of controlled trials)、中国生物医学文献数据库系统(CBM)、中国期刊全文数据库(CNKI)等收集国内外公开发表的关于早期放疗(化疗开始后30天内开始放疗)对比后期放疗(化疗开始30天以后开始放疗)治疗局限期小细胞肺癌的随机对照研究。应用统计软件Stata 11.0进行数据分析。研究人群为局限期小细胞肺癌；干预措施为胸部放疗+化疗；结局指标为2/3年死亡率和放疗相关副反应。以优势比(odds ratio, OR)及相应的95%置信区间(confidence interval, CI)作为效应指标对结局进行比较。*Q*统计量的*I*^2^检验来检测各研究间的统计学异质性。双侧*P*＜0.05认为各研究间存在明显的异质性。采用*Begg*法对发表偏倚进行量化检测。

**结果:**

最终纳入分析的文章6篇，共1 189例患者，其中早期放疗组587例，后期放疗组602例。接受早期放疗与接受后期放疗相比，两者2/3年生存优势差别无统计学意义(OR=0.78, 95%CI: 0.55-1.05, *P*=0.093)；单独分析放疗性肺炎(OR=1.93, 95%CI: 0.97-3.86, *P*=0.797)疗性食管炎(OR=1.43, 95%CI: 0.95-2.13, *P*=0.572)相关血小板减少(OR=1.23, 95%CI: 0.88-1.77, *P*=0.746)差别均无统计学意义。

**结论:**

接受早期放疗与接受后期放疗相比，2/3年生存优势、放疗相关副反应无明显区别。

## Abstract (en)

**Background and objective:**

Although evidence for a significant survival benefit of chest radiotherapy has been proven, no conclusion could be drawn regarding the optimal timing of chest radiation. The aim of this study is to explore whether the timing of chest radiation may influence the survival of the patients with limited-stage small-cell lung cancer (LS-SCLC) by performing a literature-based *meta*-analysis.

**Methods:**

By searching Medline, CENTRAL (the Cochrane central register of controlled trials), CBM, and CNKI, *et al*, we collected both domestic and overseas published documents about randomized trials comparing different timing chest radiotherapy in patients with LS-SCLC. Early chest radiation was regarded as beginning within 30 days after the start of chemotherapy. Random or fixed effect models were applied to conduct *meta*-analysis on the trials. The combined odds ratio (OR) and the 95% confidence interval (CI) were calculated to estimate the mortality in 2 or 3 years and toxicity of the two treatments. The statistical heterogeneity was determined by cochran's *Chi*-*square* test (*Q* test). The *Begg*' test was used to determine the publication bias.

**Results:**

Six trials that included a total of 1 189 patients were analyzed in the *meta*-analysis 587 patients were in the early radiation group and 602 patients were in the late radiation group. Considering all 6 eligible trials, the overall survival at 2/3 years was not significantly different between early and late chest radiation (OR=0.78, 95%CI: 0.55-1.05, *Z*=1.68, *P*=0.093). For the toxicity, no obvious difference was observed for early chest radiotherapy compared with late irradiation in pneumonitis (OR=1.93, 95%CI: 0.97-3.86, *P*=0.797), esophagitis (OR=1.43, 95%CI: 0.95-2.13, *P*=0.572) and thrombocytopenia (OR=1.23, 95%CI: 0.88-1.77, *P*=0.746), respectively.

**Conclusion:**

No statistical difference was observed in 2/3 years survival and toxicity, including pneumonitis, esophagitis and thrombocytopenia, between early radiation and late radiotherapy in LS-SCLC.

每年大约有超过120万人死于肺癌^[1]^，死亡人数超过了乳腺癌、前列腺癌和结肠癌的总和^[2]^。在所有肺癌中小细胞肺癌约占15%左右^[3]^，虽然小细胞肺癌只占小部分，但它的生物学行为特殊，预后较差。未进行任何治疗的小细胞肺癌的中位生存期只有2个月-4个月，化疗虽然可以延长患者的生存期，但长期生存率仍然很低^[4]^。近来的一项*meta*分析^[5]^结果显示化疗联合放疗较单纯化疗可以提高患者的生存率，但是何时开始实施放疗更为合适并无一致的结论^[6, 7]^。本研究采用系统评价的方法对不同放疗开始时间(早期：化疗开始后30天内；后期：化疗开始后30天以后)对局限期小细胞肺癌疗效和相关副反应方面的区别进行了定量评价，以期为临床决策提供一定的参考价值。

## 1 资料与方法

### 1.1 文献检索

通过计算机检索Medline、CENTRAL(the Cochrane central register of controlled trials)、中国生物医学文献数据库系统(CBM)、中国期刊全文数据库(CNKI)等，收集国内外公开发表的关于小细胞肺癌早期放疗对比后期放疗治疗的随机对照研究(randomized controlled trial, RCT)，检索语种为英语和汉语。以“Carcinoma, Small Cell [MeSH] AND radiotherapy [MeSH] AND Clinical trial”检索Medline、CENTRAL英文数据库；以“小细胞肺癌/小细胞肺肿瘤AND放疗/放化疗AND局限期”检索CBM、CNKI等中文数据库。为尽量避免漏查文献对入选文献的参考文献进行二次检索，相关综述、会议、摘要文章均被检索已发现可能合格的文献。同时辅以手工检索相关期刊，并用Google Scholar搜索引擎在互联网上查找相关文献。

### 1.2 纳入研究的筛选

#### 1.2.1 纳入标准

①研究设计：随机对照试验，无论是否采用盲法；②研究对象：经组织或细胞病理学确诊的局限期小细胞肺癌患者；③干预措施：胸部放疗+化疗；④结局指标：2/3年死亡率和放疗相关副反应。

#### 1.2.2 排除标准

①文献质量较差(QS＜30%)；②回顾性研究等非随机对照研究；③研究对象及干预措施不符合入选标准；④未提足够的生存资料用以计算2/3年生存率。符合以上任意一条者均予以排除。

### 1.3 纳入研究质量评价

纳入研究文献的质量按照“随机对照试验报告规范—CONSORT声明”(http://www.consort-statement.org/consort-statement/)所提供的22条标准对每一篇入选文献的质量进行量化评价。对于CONSORT声明中的每一条标准，如果入选文献中明确满足则为2分；部分满足为1分；含糊不清或未提及者则为0分。如果某条标准不适用于该研究，则该条标准不记入评分范围。如22条标准全部满足则为44分并以百分数计算(0-100%)，高分值的文献质量相对较高。对每一篇文献的质量评价采取双人平行评价的方法。

### 1.4 资料提取

采用双人平行摘录方法，由两位研究者独立阅读所获文献题目和摘要，在排除明显不符合纳入标准的试验后，对可能符合纳入标准的试验阅读全文以最终确定是否符合纳入标准。两位评价者交叉核对纳入试验的结果，对有分歧而难以确定是否纳入的试验通过讨论或由第三位评价者决定其是否纳入。提取内容包括：①一般资料：题目、作者姓名、发表日期、文献来源；②研究特征：研究对象的一般情况、各组患者的基线可比性、干预措施；③结局指标：2/3年生存率、放疗相关副反应。

### 1.5 统计分析

阅读文献，按照*meta*分析要求整理数据，建立数据库并核校数据，对数据进行定量合成。生存资料分析采用2/3年死亡率优势比(odds ratio, OR)为效应统计量；毒性反应分析采用放疗副反应发生OR为效应统计量，各效应指标均以95%置信区间(95%CI)表示，双侧*P*＜0.05认为有统计学意义。统计学异质性采用*Q*统计量的*I*^2^检验来分析。双侧*P*＞0.05认为各研究间不存在明显的异质性，采用固定效应模型(fixed effect model)合并数据；如果各研究间存在明显的异质性(*P*＜0.05)，分析其异质性的来源，对可能导致异质性的因素进行亚组分析。若两个研究组之间存在统计学异质性而无临床或方法学异质性或差异无统计学意义时，采用随机效应模型(random effect model)合并数据。如果各研究间异质性过大则不适合定量合成数据转而采用描述性分析。采用*Begg*法对发表偏倚进行量化检测。统计应用Stata 11.0统计软件完成。

## 2 结果

### 2.1 文献检索结果

初检文献143篇，阅读标题、摘要，排除不符合要求文献126篇，阅读全文排除文献11篇，最终纳入*meta*分析文献6篇^[8-13]^。共1 189例患者，其中早期放疗组587例，后期放疗组602例。试验流程如图 1。

**1:**
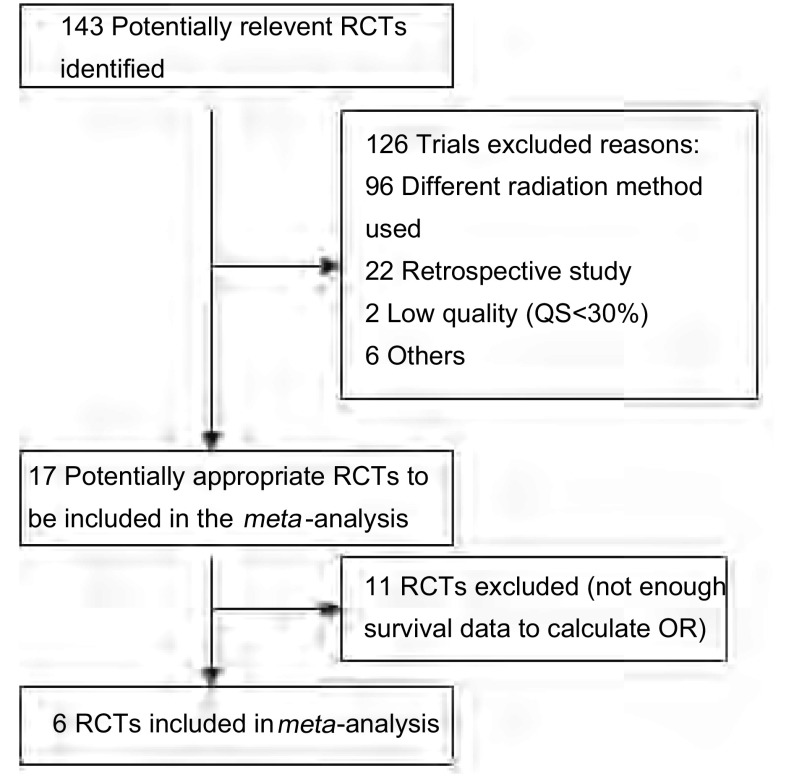
*meta*分析的流程图 Progress through the stages of the *meta*-analysis

### 2.2 纳入研究的基本特征

最终纳入研究的文献共6篇，均为英文文献(表 1)。其中放疗联合含铂方案化疗文献5篇^[8, 10-13]^，放疗联合非含铂方案化疗文献1篇^[9]^。纳入研究的6篇文献均提供了较为完整的生存资料及放疗相关副反应。根据“随机对照试验报告规范—CONSORT声明”对入选的文献进行方法学质量评价，6篇文献的平均得分为65.5%(64%-70%)。

**1:** 纳入研究的基本特征 General characteristics of included trials

Trial	No. of patients	Timing (Early/Late)	OS (%)	Age range	PS	2/3 year survival rate	OR	95%CI
Murry (1993)	155	E	68	NR	ECOG 0-1:87%	40%	0.77	0.49-1.23
	153	L		NR	ECOG 0-1:90%	34%		
Jeremic (1997)	52	E	64	40-67	KPS 90-100:52%	71%	0.46	0.20-1.03
	51	L		44-66	KPS 90-100:47%	53%		
Work (1997)	99	E	66	36-70	KPS 80-100:82%	20%	0.87	0.43-1.76
	100	L		36-69	KPS 80-100:80%	18%		
Perry (1998)	125	E	70	32-79	ECOG 0-1:86%	18%	1.97	1.10-3.53
	145	L		32-79	ECOG 0-1:87%	30%		
Skarlos (2001)	42	E	58	40-76	ECOG 0-1:76%	36%	0.71	0.28-1.81
	39	L		38-79	ECOG 0-1:85%	28%		
Takada (2002)	114	E	67	39-74	ECOG 0-1:95%	54%	0.45	0.27-0.77
	114	L		30-74	ECOG 0-1:95%	35%		

### 2.3 各独立研究结果的异质性检验

纳入研究的6篇文献分别以2/3年死亡优势比OR、放疗副反应优势比OR为效应指标，采用*I*^2^统计量进行异质性检验。异质性检验结果：2/3年死亡优势比OR效应指标，各研究间存在异质性；放疗性肺炎、放疗性食管炎及放疗相关血小板减少优势比OR为效应指标，各研究间均不存在异质性(表 2)。

**2:** 各研究结果的异质性检验 Test(s) of heterogeneity in the included trials

Effect size	*Chi*-*square*	*I*-*square*	*P*	Freedom
2/3 year survival	13.75	63.6%	0.017	5
Pneumonitis	1.02	0.0%	0.797	3
Esophagitis	2.91	0.0%	0.572	4
Thormbocytopenia	2.70	0.0%	0.746	5

### 2.4 数据定量合成

#### 2.4.1 生存分析

纳入研究的6篇文献均提供了较为完整的生存资料，2/3年生存率早期放疗组39.8%，后期放疗组为33.0%。*meta*分析的结果显示，接受早期放疗与接受后期放疗相比两者2/3年生存优势差别无统计学意义(OR=0.78, 95%CI: 0.55-1.05, *Z*=1.68, *P*=0.093)(图 2)。

**2:**
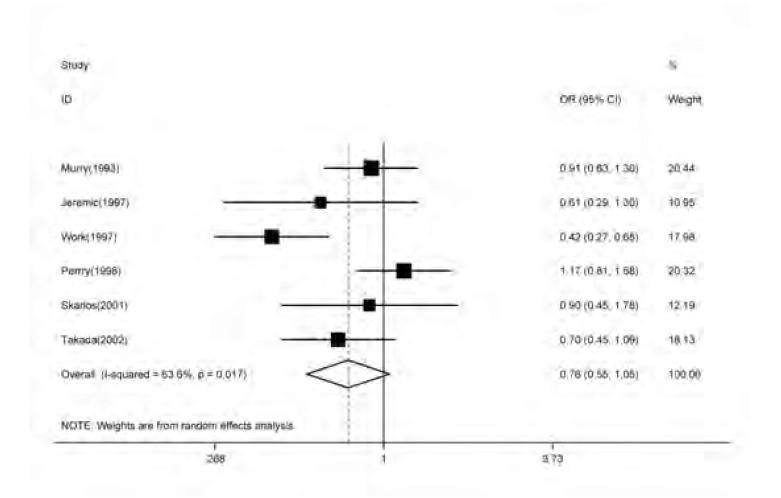
早期对比后期放疗治疗局限期小细胞肺癌2/3年生存优势的森林图 Forest plot of survival as a function of the timing of chest radiation for 2/3 year survival

#### 2.4.2 放疗副反应

根据国际癌症联合会制定的标准(http://ctep.cancer.gov/protocolDevelopment/electronic_applications/docs/ctcaev3.pdf)，4篇文献均提供了放疗性肺炎的发生率^[9-12]^；5篇文献提供了放疗性食道炎的发生率^[9-13]^；6篇文献均提供了放疗性血小板减少的发生率。总体来看早期放疗患者更易出现放疗相关副反应(OR=1.37, 95%CI: 1.08-1.74, *Z*=2.58, *P*=0.010)；单独分析相关副反应，接受早期放疗的患者与接受后期放疗的患者相比，在放疗性肺炎、放疗性食管炎及放疗相关血小板减少方面差别均无统计学意义。接受早期放疗者有发生更多相应并发症的趋势：放疗性肺炎(OR=1.93, 95%CI: 0.97-3.86, *Z*=1.87, *P*=0.061)；放疗性食管炎(OR=1.43, 95%CI: 0.95-2.13, *Z*=1.73, *P*=0.083)；放疗相关血小板减少(OR=1.23, 95%CI: 0.88-1.71, *Z*=1.20, *P*=0.232) (图 3)。

**3:**
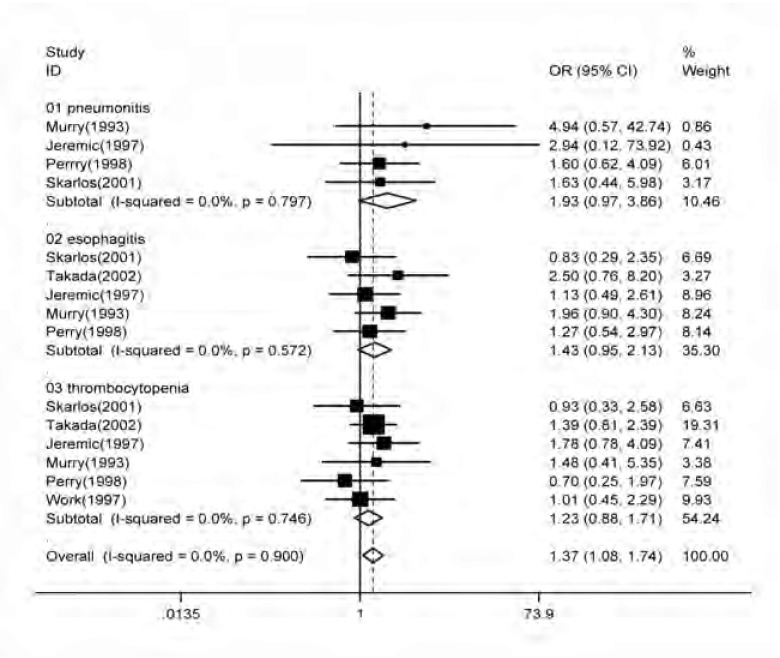
早期对比后期放疗治疗局限期小细胞肺癌副反应发生优势的森林图 Forest plot of toxicity as a function of the timing of chest radiation

### 2.5 发表性偏倚的识别及敏感性分析

*Begg*法量化检测发表偏倚，*Begg*’test中Pr＞|*z*|=0.452＞0.05，漏斗图中各点沿中间水平线两侧均匀分布，全部位于预计95%CI内(图 4)，检测结果提示不存在发表偏倚。对纳入研究的6篇文献进行敏感性分析，剔除Murry^[12]^、Jeremic^[10]^、Skarlos^[11]^、Takada^[13]^、Work^[8]^中的任意一篇文献后*meta*分析的OR均位于0.68-0.77之间，95%CI: 0.48-1.14(包括1在内)，剔除前后未发生明显变化，结论的性质未发生改变；当剔除Perry^[9]^的研究后发现OR值变为0.68，且95%CI: 0.49-0.93(结果不包括1在内)，剔除前后发生明显变化，结论的性质也随着发生改变，由剔除前的差别无统计学意义变为剔除后的接受早期放疗者2/3年生存率存在优势，提示Perry的研究是文章异质性的主要来源(图 5)。

**4:**
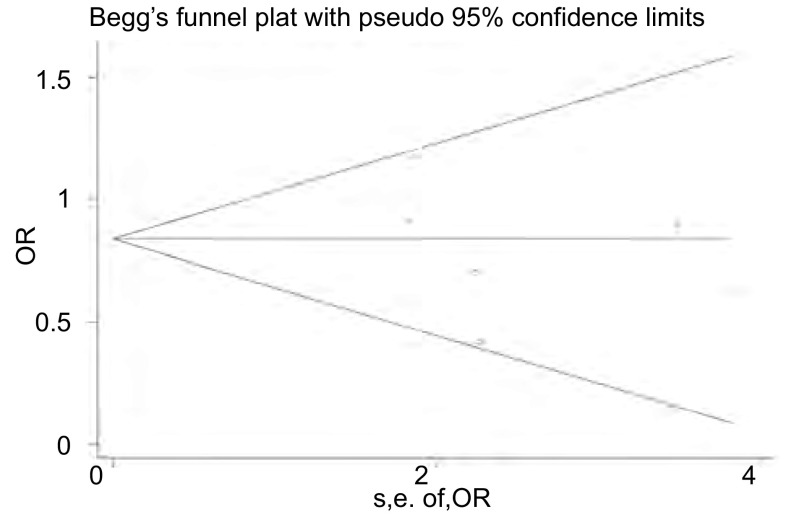
*Begg*法检测发表偏倚的漏斗图 The funnel plot for *Begg*'s test

**5:**
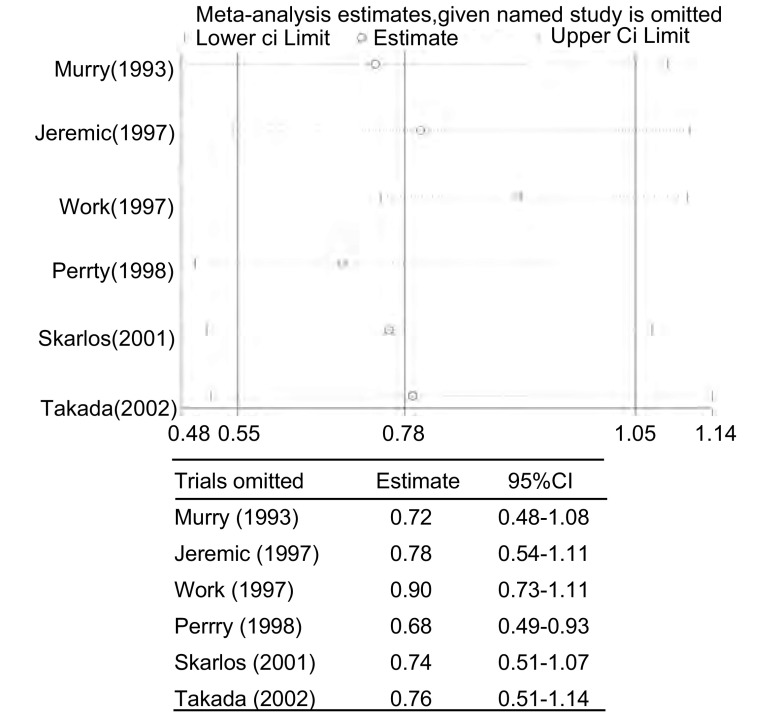
各篇文献对*meta*分析结果的影响 The influence of each trial for the outcome of the *meta*-analysis

## 3 讨论

*meta*分析结果显示放疗联合化疗可以提高局限期小细胞肺癌生存率^[14]^，但化疗开始后何时开始实施放疗仍然存在争议^[7, 14]^。已有研究^[15, 16]^证实，在很多实体肿瘤中，肿瘤细胞在接触细胞毒性药物后30天左右开始加速克隆增殖。基于此，我们进行了该项关于早期放疗(化疗开始后30天内)对比后期放疗治疗局限期小细胞肺癌的*meta*分析。该研究结果并未证实早期放疗与后期放疗在2/3年生存率方面不同；但接受早期放疗患者会出现更多的总的放疗相关副反应(OR=1.37, 95%CI: 1.08-1.74, *Z*=2.58, *P*=0.010)。单独分析放疗性肺炎、放疗性食管炎及放疗相关血小板减少方面副反应，差别均无统计学意义，只观察到接受早期放疗者有发生更多相应并发症的趋势。

该*meta*分析只进行了某个时点(2/3年)早期放疗与后期放疗患者生存差别比较，并未对治疗随访期间患者的整体生存曲线做比较，因此并不能说明早期放疗与后期放疗对患者整体生存影响无差别，需要更多的数据做进一步细致的分析。接受早期放疗患者易出现更多的总的放疗相关副反应(OR=1.37, 95%CI: 1.08-1.74, *Z*=2.58, *P*=0.010)，可能是由于放疗开始时间距离化疗时间较近，放疗与化疗副反应的叠加与协同作用所造成的。2/3年生存率方面，入选的各项研究间存在统计学异质性(χ^2^=13.75, *I*^2^=63.3%, *P*=0.017)，敏感性分析提示统计学异质性的来源为Perry的研究，同时Perry的研究与其它5项研究之间也存在一定的临床异质性，Perry的研究为放疗联合非含铂方案化疗而其它5项研究则为放疗联合含铂方案化疗。虽然本研究采用了随机效应模型对数据进行整合分析，用以调整统计学异质性对结论的影响，但是临床异质性是不大可能通过统计学调整而消除的。当我们将Perry的研究剔除后发现该*meta*分析结论的性质出现了改变，早期放疗与后期放疗在2/3年生存率方面不同，且有统计学意义(OR=0.69, 95%CI: 0.56-0.85, *Z*=3.41, *P*=0.001)。Perry的研究对于结论的影响较为重要，它直接影响结论的性质，说明该*meta*分析结论的稳定性较差，因此需要更多的临床随机对照研究来加强结论的稳定性。

## References

[1] Ginsberg MS (2005). Epidemiology of lung cancer. Semin Roentgenol.

[2] Schiller JH, Harrington D, Belani CP (2002). Comparison of four chemotherapy regimens for advanced non-small-cell lung cancer. N Engl J Med.

[3] Molina JR, Yang P, Cassivi SD (2008). Non-small cell lung cancer: epidemiology, risk factors, treatment, and survivorship. Mayo Clin Proc.

[4] Kelly K (2000). New chemotherapy agents for small cell lung cancer. Chest.

[5] Pignon JP, Arriagada R, Ihde DC (1992). A *meta*-analysis of thoracic radiotherapy for small-cell lung cancer. N Engl J Med.

[6] Kumar P (1997). The role of thoracic radiotherapy in the management of limitedstage small cell lung cancer: past, present, and future. Chest.

[7] De Ruysscher D, Vansteenkiste J (2000). Chest radiotherapy in limited-stage small cell lung cancer: facts, questions, prospects. Radiother Oncol.

[8] Work E, Nielsen OS, Bentzen SM (1997). Randomized study of initial versus late chest irradiation combined with chemotherapy in limited-stage small-cell lung cancer. Aarhus Lung Cancer Group. J Clin Oncol.

[9] Perry MC, Hemdon JE 3rd, Eaton WL (1998). Thoracic radiation therapy added to chemotherapy for small-cell lung cancer: an update of Cancer and Leukemia Group B Study 8083. J Clin Oncol.

[10] Jeremic B, Shibamoto Y, Acimovic L (1997). Initial versus delayed accelerated hyperfractionated radiation therapy and concurrent chemotherapy in limited small-cell lung cancer: a randomized study. J Clin Oncol.

[11] Skarlos DV, Samantas E, Briassoulis E (2001). Randomized comparison of early versus late hyperfractionated thoracic irradiation concurrently with chemotherapy in limited disease small-cell lung cancer: a randomized phase Ⅱ study of the Hellenic Cooperative Oncology Group (HeCOG). Ann Oncol.

[12] Murray N, Coy P, Pater JL (1993). Importance of timing for thoracic irradiation in the combined modality treatment of limited-stage small-cell lung cancer. The National Cancer Institute of Canada Clinical Trials Group. J Clin Oncol.

[13] Takada M, Fukuoka M, Kawahara M (2002). Phase Ⅲ study of concurrent versus sequential thoracic radiotherapy in combination with cisplatin and etoposide for limited-stage small-cell lung cancer: results of the Japan Clinical Oncology Group Study 9104. J Clin Oncol.

[14] Warde P, Payne D (1992). Does thoracic irradiation improve survival and local control in limited-stage small-cell carcinoma of the lung? A *meta*-analysis. J Clin Oncol.

[15] Davis AJ, Tannock JF (2000). Repopulation of tumour cells between cycles of chemotherapy: a neglected factor. Lancet Oncol.

[16] Wu L, Tannock IF (2003). Repopulation in murine breast tumors during and after sequential treatments with cyclophosphamide and 5-fluorouracil. Cancer Res.

